# Guanidinoacetic acid deficiency: a new entity in clinical medicine?

**DOI:** 10.7150/ijms.47757

**Published:** 2020-09-12

**Authors:** Sergej M. Ostojic, Laszlo Ratgeber, Andras Olah, Jozsef Betlehem, Pongras Acs

**Affiliations:** 1 FSPE Applied Bioenergetics Lab, University of Novi Sad, Novi Sad, Serbia.; 2 Faculty of Health Sciences, University of Pecs, Pecs, Hungary.

**Keywords:** guanidinoacetic acid, AGAT deficiency, renal failure, epilepsy, traumatic injury

## Abstract

Guanidinoacetic acid (GAA, also known as glycocyamine or betacyamine) is a naturally-occurring derivative of glycine and a direct metabolic precursor of creatine, a key player in high-phosphate cellular bioenergetics. GAA is found in human serum and urine, with circulating GAA likely reflects an equilibrium between its endogenous production and utilization/excretion. GAA deficiency (as indicated by low serum GAA) has been reported in various conditions yet this intriguing clinical entity appears to be poorly characterized as yet, either as a primary deficit or a sequel of secondary disease. This minireview article summarizes the inherited and acquired disorders with apparent GAA deficiency and discusses a possible relevance of GAA shortfall in clinical medicine.

## Introduction

**Figure 1:**
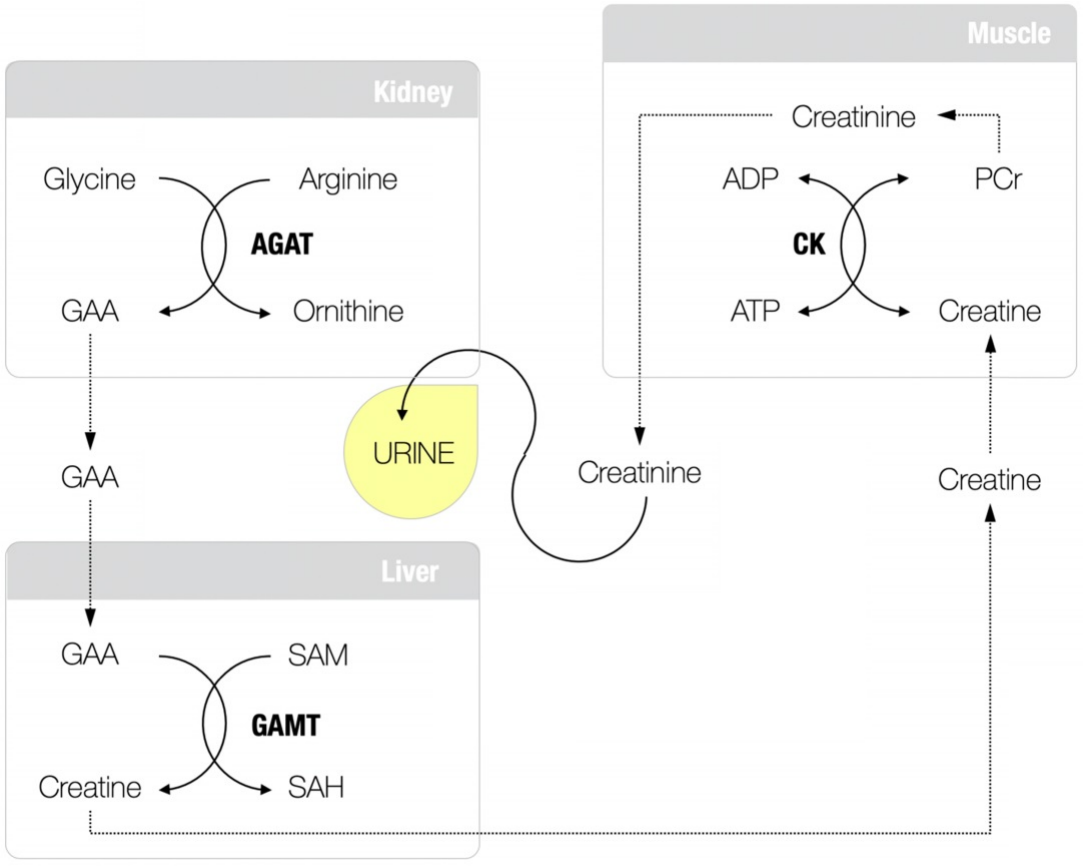
Guanidinoacetic acid (GAA) and creatine metabolism. AGAT, *L*-arginine:glycine amidinotransferase; SAM, *S*-adenosylmethionine; GAMT, guanidinoacetate *N*-methyltransferase; SAH, *S*-adenosylhomocysteine; CK, creatine kinase; PCR, phosphocreatine; ADP, adenosine diphosphate; ATP, adenosine triphosphate.

Guanidinoacetic acid (GAA; molecular formula: C_3_H_7_N_3_O_2_) is an amino acid-like compound and relatively unexplored, although long-known, human metabolite. About 85 years ago, Dr. Clarence J. Weber from the University of Kansas School of Medicine was arguably the first who isolated GAA (also known as glycocyamine) from the urine of two patients with pseudo-hypertrophic muscular dystrophy and the urine of normal humans collected during the period of fasting 1. This seminal work advances GAA as a regular constituent of normal urine (and serum), and its presence has been implied not to be a result of its intake from the food but rather an intermediary product in creatine metabolism (**Figure 1**), a fact confirmed in further studies 2-4. GAA is produced from conditionally essential amino acids glycine and arginine through the reaction catalyzed by *L*-arginine:glycine amidinotransferase (AGAT; EC 2.1.4.1), the most likely control enzyme in the pathway and the committed step in the formation of creatine 5. The reaction catalyzed by AGAT takes place mainly in the kidney and pancreas (also the liver, brain, endocrine tissues, and gut), with GAA renal synthesis may account for ≥ 20% of total GAA production 6. Newly synthesized GAA is released to the circulation to be subsequently taken up by the liver (also the brain, myocardium, reproductive organs) for the second step in creatine biosynthesis catalyzed by guanidinoacetate *N*-methyltransferase (GAMT; EC 2.1.1.2). GAMT transfers the methyl group from *S*-adenosylmethionine to GAA to form creatine and *S*-adenosylhomocysteine. Creatine is finally discharged into the bloodstream from where it can be absorbed by miscellaneous energy-demanding tissues, while residual GAA likely been excreted via the kidney 7.

**Figure 2:**
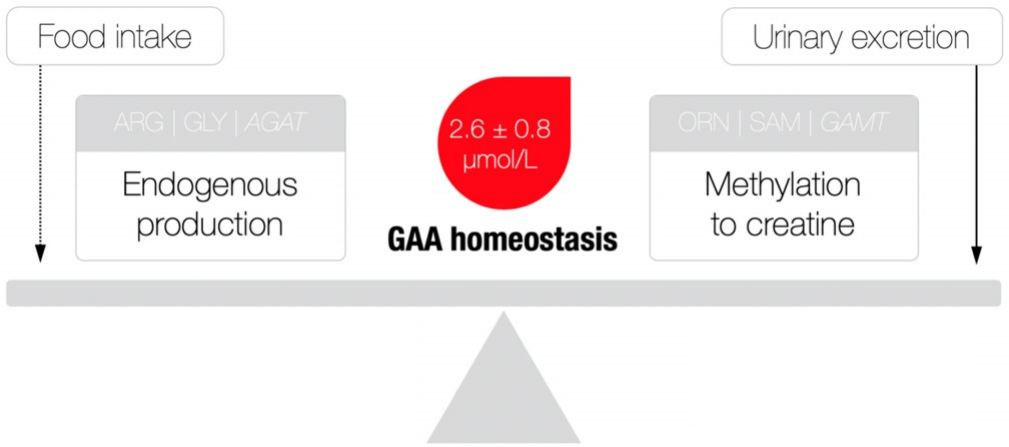
A theoretical model of guanidinoacetic acid (GAA) homeostasis. AGAT, *L*-arginine:glycine amidinotransferase; ARG, arginine; GLY, glycine; ORN, ornithine; SAM, *S*-adenosylmethionine; GAMT, guanidinoacetate *N*-methyltransferase. A dotted line indicates a minimal contribution to the model. Reference values for serum GAA were derived from 24.

Besides serving as a direct precursor of creatine, GAA appears to have no other major physiological roles 8, although exogenous GAA may stimulate hormonal release and neuromodulation, alter metabolic utilization of amino acids (*e.g.*, arginine, creatine), and act as an oxidant-antioxidant tuner 9. Having in mind its rather simple metabolic fate, GAA homeostasis (as indicated by the levels of GAA in the circulation) perhaps illustrates the balance between endogenous production/utilization and kidney excretion, due to the fact that the amount of GAA from food sources is negligible (~ 10 mg/kg of meat) 10. That being said, a possible deficiency could be attributable to either compromised synthesis (that may include the shortage of building blocks of glycine and arginine, AGAT malfunction and the parent organ failure) and/or enhanced consumption/elimination of GAA (**Figure 2**), with GAA deficiency perhaps singled out as a novel clinical entity. Although this member of the guanidine family has been identified as a naturally occurring compound in the human biofluids way back to the 1930s, GAA deficiency actually remains poorly addressed in experimental and clinical medicine up to this point. Here, we summarize inherited and acquired disorders characterized by GAA deficiency, and discuss the possible clinical relevance of this deficit; only human studies that provided data for circulating GAA concentrations were included in this analysis after a detailed search of published papers from scientific journals indexed in PubMed, Scopus, and Web of Science. This includes kidney and thyroid dysfunctions, neurological diseases and post-traumatic GAA deficit, along with *L*-arginine:glycine amidinotransferase deficiency**,** ornithine aminotransferase deficiency and urea cycle disorders; we also put forward criteria for normal and abnormal GAA levels, and clinical features of GAA deficiency.

## Kidney diseases

Since the kidney is among the main organs of GAA synthesis, any persistent and severe renal disorder could jeopardize GAA output and instigate GAA deficiency. Sawynok and Dawborn 11 were among the first authors who reported low plasma GAA levels (< 2 μmol/L) in both ambulatory and hospitalized uremic patients (*e.g.* blood urea ranging from 62 to 325 mg/100 mL) and patients undergoing maintenance hemodialysis. Tofuku and co-workers 12 found impaired metabolism of GAA in uremia, with renal GAMT activity reduced for ~ 71% as serum urea nitrogen level rises in the course of renal damage, resulting in lower concentration of serum GAA in patients with chronic renal failure as compared to normal subjects (2.4 ± 0.1 μmol/L* vs.* 1.5 ± 0.1 μmol/L). Similar results were found in non-dialyzed men and women with chronic renal insufficiency 13, with a drop in serum GAA positively correlated with the progression of renal disease. For instance, a subsample of male patients with more severe renal damage (*e.g.* creatinine clearance, CCR < 10 mL/min) experienced the most prominent reduction (~ 49.8 %) in circulating GAA as compared to control group (2.6 ± 0.5 μmol/L *vs.* 1.4 ± 0.6 μmol/L). A Japanese group 14 corroborated above findings and reported that serum GAA decreased with loss of renal function in patients with chronic glomerulonephritis (CGN), particularly in those with CCR below 30 mL/min (*e.g.* from 3.3 μmol/L in healthy adults to 2.1 μmol/L in CGN patients). The authors also demonstrated that patients with nephropathy induced by non-insulin dependent diabetes mellitus (DM) experienced a significant drop in circulating GAA, with a degree of GAA deficit CCR-dependent, and the authors implied that GAA deficiency might be a reason of muscle wasting in this clinical population. Besides diminished production, GAA deficiency in renal failure could be aggravated by augmented GAA urinary excretion perhaps as a consequence of defective transmethylation extrarenally, with children with nephrotic syndrome encountered a tremendous increment (~ 900%) in 24 h urinary excretion of GAA 15. However, other studies found no increased excretion of GAA in kidney disease, except in a subsample of patients with DM-related nephropathy and CCR > 60 mL/min 14.

## Thyroid dysfunction

A group led by Dr. Bart Marescau from the University of Antwerp analyzed circulating guanidino compounds in a large group of patients with common thyroid gland disorders 16. The authors found a highly significant reduction in serum GAA in patients with subclinical hyperthyroidism (for 16.7% on average) and clinical hyperthyroidism (36.8%), suggesting thyroid dysfunction-driven GAA deficit that could advance the use of serum GAA as an additional sensitive marker of hyperthyroidism. The authors were not able to address the possible mechanism(s) for this phenomenon yet the level of guanidino compounds (including GAA) appears to be influenced by thyroid hormones by the way of their action on the skeletal muscle, kidney, liver, and peripheral protein metabolism 17,18. Besides, human thyroid tissue appears to contain quantifiable amounts of guanidino compounds *per se* 19,20 with tissue GAA turnover might be altered in the gland malfunction. Hypothetically, GAA deficiency found in thyroid dysfunction might be due to the fact that the level of thyroid function alters the metabolism of steroid hormones, including their rates of production and catabolism in the liver and other tissues 21, driving steroids-related changes in creatine metabolism 22.

## Neurological diseases

The first case of GAA deficiency in common neurological disorders was described almost 30 years ago. Shiraga and co-workers 23 examined the serum levels of guanidino compounds in 58 neurological male and female patients with cerebral palsy, encephalomeningitis, intracranial hemorrhage, and other conditions. The authors found that the average serum levels of GAA in both non-epileptic and epileptic patients were significantly lower than in healthy controls (3.2 μmol/L *vs.* 2.1 μmol/L) which perhaps implies a hypoactive GAA metabolism in neurological patients. In addition, circulating GAA appears to be less reduced in a subpopulation of epileptic patients treated with appropriate medication as compared to serum GAA levels found in patients with uncontrolled epilepsy (2.2 μmol/L *vs.* 1.6 μmol/L). These findings suggest that common medicines given to neurological patients might improve GAA turnover and tend to restore normal GAA equilibrium that could contribute to the overall benefit. However, not all human trials found GAA deficiency in neurological patients. A recent study reported that plasma GAA concentrations in 9 patients suffering from acute stroke or transitory ischemic attacks were comparable to those measured in healthy humans 24. A possible discrepancy might be due to the nature of neurological disorders evaluated in those studies (acute *vs.* chronic pathologies) that might alter GAA homeostasis in a dissimilar manner.

## Post-traumatic GAA deficiency

An interesting study from Manchester University Medical School 25 evaluated changes in plasma guanidino compounds in 31 patients who suffered moderate or severe accidental injuries (e.g. fracture, head trauma, abdominal injury, compartment syndrome). Besides other findings, the authors reported that most patients encountered low plasma GAA concentrations after an accident, with plasma GAA declined to a very low level of 0.3 μmol/L in a case with renal trauma (female, 17 years; injury severity score 34 out of 75). Normal levels returned with the restoration of kidney function after 15 periods of hemodialysis over 17 days. The mechanisms for this trauma-driven GAA deficit remain undisclosed. However, the fact that traumatic injury could negatively affect kidney function 26, and perhaps deprive the organ of normal blood flow and/or induce a pro-inflammatory phenotype and cellular dysfunction or apoptotic death 27 may account for GAA deficiency, as the kidneys are the primary organs of GAA synthesis.

## *L*-arginine:glycine amidinotransferase deficiency

AGAT deficiency (also known as Creatine Deficiency Syndrome-3) is a very rare inherited disorder of creatine metabolism, characterized by the lack of GAA-synthesizing enzyme, *L*-arginine:glycine amidinotransferase. The syndrome primarily affects the brain and skeletal muscle, and patients with AGAT deficiency have mild to moderate pathology that includes mental retardation, developmental delay, muscle weakness and epilepsy 28,29. Levels of GAA in plasma are particularly low (0.01 - 0.04 μmol/L), as well as urinary GAA (2.4 - 5.8 μmol/L; reference values = 311 ± 191 μmol/L) 30. Less than 20 patients with AGAT deficiency have been identified globally 31 so this clinical syndrome remains incompletely described so far.

## Ornithine aminotransferase deficiency

Another inherited disease with pertinent GAA shortfall is ornithine aminotransferase (OAT) deficiency. This rare inborn error of ornithine metabolism (also known as gyrate atrophy of the choroid and retina) is caused by decreased activity of ornithine aminotransferase, an enzyme that is involved in the formation of proline from ornithine. OAT deficiency is clinically characterized by myopia which progresses to night blindness, subcapsular cataract and elevated levels of ornithine in the blood 32. Since creatine synthesis requires the conversion of arginine and glycine into GAA and ornithine, pathological ornithine accumulation (650 μmol/L to 1.3 mmol/L) can inhibit AGAT and subsequently block GAA production (and creatine biosynthesis) contributing to disease features 33. Valayannopoulos and co-workers 34 confirmed decreased levels of GAA in plasma (and urine) of 7 patients with OAT deficiency aged from 11 to 27 years. Various neurocognitive impairments, including mild to severe mental retardation, educational difficulties, major visuospatial dyspraxia, aggressive behavior, and epilepsy, were found in all OAT patients, with plasma and urine GAA levels were ≤ 0.61 μmol/L and 4 μmol/mmol cr, respectively.

## Urea cycle disorders

Urea cycle disorders (UCDs) results from inherited deficiencies in any one of the six enzymes or two transporters of the urea cycle pathway, with the clinical presentation and severity influenced by the importance of the defective compound (for a detailed review see Ref. 35). Typically characterized by hyperammonemia, loss of appetite, vomiting, lethargy, and behavioral abnormalities, distinct types of UCDs can impede GAA synthesis due to the overproduction of ornithine (presented as hyperornithinemia) and concomitant inhibition of AGAT. Arias and co-workers 36 analyzed 15 patients with various UCDs (age range from 2 days to 2 years) and showed significantly lower plasma GAA concentrations when compared with age-matched controls (0.77 μmol/L *vs.* 1.7 μmol/L). The authors advanced the use of plasma GAA as a practical and relevant parameter to consider in the follow-up of patients with UCDs.

## Other conditions

Besides the above pathologies, a transient or long-standing GAA deficiency becomes evident in several non-clinical populations, including elderly or healthy men and women exposed to heavy exercise. Matsumura and co-workers 37 examined the metabolism of guanidino compounds in middle-aged and elderly subjects by measuring serum concentrations of several specific guanidines. The authors reported that the elderly subjects tended to have lower serum GAA levels than middle-aged subjects (2.3 μmol/L *vs.* 3.2 μmol/L), with bedridden elderly had even lower GAA levels (accompanied by lighter muscle mass) than their ambulatory peers (1.8 μmol/L *vs.* 2.8 μmol/L). This might be due to age-dependent suppression of AGAT activity modulated by dietary and hormonal factors 8, resulting in decreased GAA production in the elderly subjects. Furthermore, a transitory exercise-generated drop in serum GAA (for approx. 20.5% on average) has been found after a session of exhaustive cycling 38, strenuous running 39 and heavy resistance exercise to exhaustion 40. A heavy exercise-induced GAA depletion could be attributable to an exercise-induced reduction in renal hemodynamics that might reduce the availability of glycine and arginine or by suppressing the activity of AGAT 39. Heavy exercise might also provoke ample methylation of GAA to creatine, the main source of energy for high-intensity exercise 41 which inevitably causes GAA deficiency.

## Normal serum GAA levels

The normal levels of circulating GAA in healthy humans are evaluated in a handful of studies. The most recent trial known to the author reported plasma GAA for healthy subjects to be 2.6 ± 0.8 μmol/L 24. The values reported in this study are close or equivalent to those published for healthy men and women by other research groups in plasma and serum 13,23,41-43, and thus could be considered normal readings usually found in healthy adults. Almeida and co-workers 28 reported values for plasma GAA in 60 healthy subjects (age range: newborns to 90 years) who had no metabolic, renal or neurological disorders, with reference ranges displayed as age-specific (e.g. children aged 0-15 years *vs.* subjects older than 15 years). Unfortunately, no means or variation indices were presented in this study, although children tended to show lower plasma GAA levels as compared to their older counterparts while no significant difference was observed between genders. Another study 44 reported rather high levels of serum GAA in healthy adults and children, with the values in plasma and from blood spots (filter paper) approximately 90% higher as compared to other published data. Differences across studies for GAA reference values could be due to various methods used for GAA determination that might interfere with GAA separation (e.g. HPLC with post-column derivatization *vs.* stable‑isotope dilution GC-MS measurement). This highlights the need to establish a gold-standard protocol to evaluate serum or plasma GAA in laboratory medicine. For the time being, a serum GAA < 0.04 μmol/L is highly suggestive of severe GAA deficiency, as seen in AGAT defect 30 while marginal GAA deficiency perhaps identified when serum GAA values drop below 2.0 μmol/L 16.

Although GAA and creatine metabolism are tightly interconnected, GAA deficiency and low serum GAA does not go along with creatine deprivation on every occasion. As an example, a reduced circulating GAA level is accompanied by normal serum creatine in patients with chronic renal insufficiency 13, or by high creatine in the blood of patients with accidental trauma 25 and hyperthyroidism 16. This perhaps illustrates a distinct metabolic fate of each compound in specific pathologies and opens a possibility for using serum GAA as an independent disorder-specific biomarker. In addition, low serum GAA might be more sensitive than the conventional renal function markers in evaluating renal failure in primary or secondary GAA deficiency that affect the kidneys 45.

## Clinical features of GAA deficiency

Either as a disorder that could be due to a root cause of GAA shortage (e.g. AGAT deficiency) or triggered by a condition that provokes GAA diminution (such as trauma-driven kidney failure), GAA deficiency likely entails clinical attributes of low levels of energy output due to its role in energy metabolism 8. The organs with the highest demands for cellular energy are the brain, myocardium and skeletal muscle. Therefore, muscular findings in GAA deficiency could include generalized weakness, tiredness and reduced work capacity, while neurological signs and symptoms involve brain fatigue, intellectual disability, behavior and movement disorders, with the features perhaps progress as GAA deficit being more notable 16, or being more severe owing to the early age at which the shortage begins 29. Even so, clinician- and patient-reported outcomes remain largely non-specific and determining serum GAA is necessary to confirm GAA deficiency. Additional approaches that can help expanding clinical features of GAA deficiency may include the analysis of GAA concentrations in saliva 46 or in the muscle and brain using magnetic resonance spectroscopy 47,48.

## Future directions for research

Many issues on GAA deficiency need to be addressed before recognizing it as an authentic clinical entity. First and foremost, more studies are needed to define the reference ranges for GAA deficiency in various clinical disorders. The largest population study so far described the GAA reference values in a population of 6334 French patients with neurological symptoms 49, with sex- and age-specific laboratory values shown for plasma and urinary GAA. Second, only a few studies described a variation in serum GAA across different stages of a specific disease, with chronic glomerulonephritis perhaps characterized more appropriate than other conditions in terms of GAA deficiency with the progression of the disease 14. It also remains open to question does the inability of the kidney to synthesize GAA in a GAA deficiency with renal failure component could be compensated by other GAA-producing organs 12; we have to trace GAA deficiency in comorbid conditions that could tackle GAA homeostasis in multiple aspects as well. Likewise, does the low availability of arginine and glycine negatively affect GAA output and contribute to the disease remain unknown at the moment, particularly for conditions such as arginine deficiency syndrome 50, cerebral inflammation 51, or hypoglycinemia-related disorders 52. Finally, the possible links between GAA deficiency and amplified GAA urinary excretion have yet to be analyzed.

## Conclusion

GAA deficit has been found in many pathologies, from very rare creatine deficiency syndrome and urea cycle defects to more common conditions, including traumatic injuries, chronic kidney disease, and neurological disorders. Characterized by the reduced serum GAA, GAA deficiency seems to reflect a disbalance between its endogenous production and utilization/excretion in disorders that primarily affect GAA-pivotal organs, such as the kidney, liver and skeletal muscle. Due to somewhat divergent specificity to creatine turnover, GAA deficiency might be considered as a unique metabolic feature in the follow-up of various diseases. To validate its possible clinical significance, additional well-sampled studies are warranted to describe GAA dynamics in large cohorts of patients and evaluate possible GAA deficiency features across the continuum of GAA values and conditions.

## Abbreviations

ADP: adenosine diphosphate
AGAT: *L*-arginine glycine amidinotransferase
ARG: arginine
ATP: adenosine triphosphate
CCR: creatinine clearance
CGN: chronic glomerulonephritis
CK: creatine kinase
DM: diabetes mellitus
GAA: guanidinoacetic acid
GAMT: guanidinoacetate *N*-methyltransferase
GLY: glycine
OAT: ornithine aminotransferase
ORN: ornithine
PCR: phosphocreatine
SAH: *S*-adenosylhomocysteine
SAM: *S*-adenosylmethionine
UCDs: urea cycle disorders
